# Hydrogen evolution from water catalyzed by cobalt-mimochrome VI*a, a synthetic mini-protein†
†Electronic supplementary information (ESI) available: UV-vis spectra, mass spectra, additional electrochemical experiments, tables. See DOI: 10.1039/c8sc01948g


**DOI:** 10.1039/c8sc01948g

**Published:** 2018-09-14

**Authors:** Vincenzo Firpo, Jennifer M. Le, Vincenzo Pavone, Angela Lombardi, Kara L. Bren

**Affiliations:** a Department of Chemical Sciences , University of Naples Federico II , Complesso Universitario Monte S. Angelo , via Cintia 45 , 80126 Naples , Italy . Email: alombard@unina.it; b Department of Chemistry , University of Rochester , Rochester , NY 14627 , USA . Email: bren@chem.rochester.edu

## Abstract

A synthetic enzyme is reported that electrocatalytically reduces protons to hydrogen (H_2_) in water near neutral pH under aerobic conditions. Cobalt mimochrome VI*a (CoMC6*a) is a mini-protein with a cobalt deuteroporphyrin active site within a scaffold of two synthetic peptides covalently bound to the porphyrin. Comparison of the activity of CoMC6*a to that of cobalt microperoxidase-11 (CoMP11-Ac), a cobalt porphyrin catalyst with a single “proximal” peptide and no organized secondary structure, reveals that CoMC6*a has significantly enhanced longevity, yielding a turnover number exceeding 230 000, in comparison to 25 000 for CoMP11-Ac. Furthermore, comparison of cyclic voltammograms of CoMC6*a and CoMP11-Ac indicates that the trifluoroethanol-induced folding of CoMC6*a lowers the overpotential for catalytic H_2_ evolution by up to 100 mV. These results demonstrate that even a minimal polypeptide matrix can enhance longevity and efficiency of a H_2_-evolution catalyst.

## Abstract 2

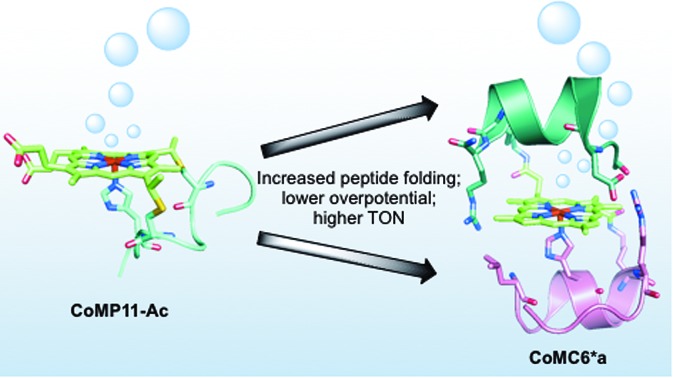
The folding of a synthetic mini-hydrogenase is shown to enhance catalyst efficiency and longevity.

## Introduction

Organisms have evolved enzymes that catalyze selective multielectron, multiproton reactions using earth-abundant metals, inspiring the development of related complexes and assemblies for energy-related catalysis.^1^–^3^ Hydrogenase enzymes catalyze reversible proton reduction to H_2_ in mild conditions with low (or no) overpotential.^4^–^6^ The efficiency of these enzymes is ascribed to the protein matrix, which provides a defined environment for the active site to tune reduction potential, provide second-sphere interactions, and transfer electrons and protons.^4^,^7^,^8^ Synthetic H_2_-evolution electrocatalysts have been reported with second-sphere interactions or proton shuttling sites proposed to lower overpotentials; most require organic solvents or water-solvent mixtures,^9^–^12^ but a few function in water.^13^–^15^

**Fig. 1:**
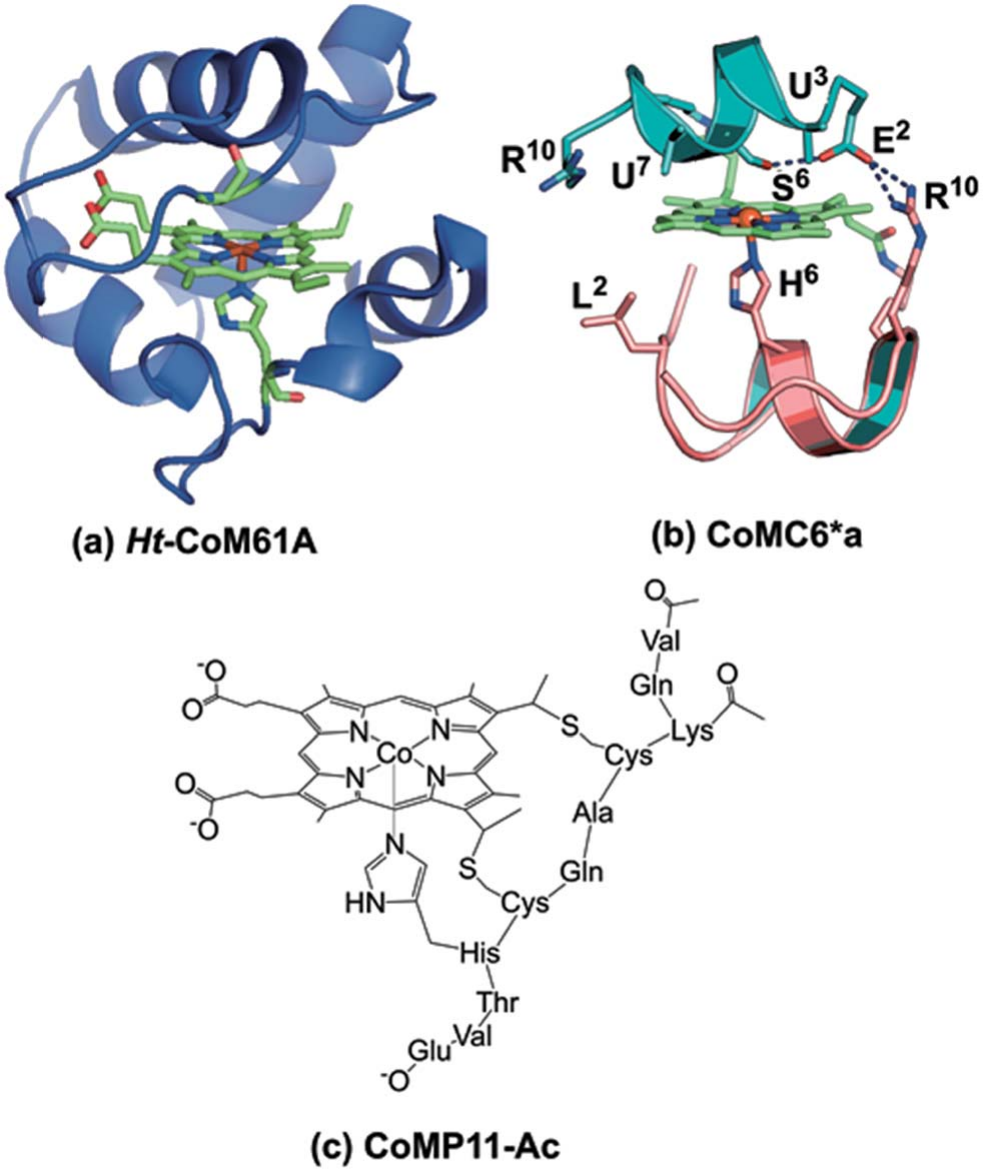
(a) Model of *Ht*-CoM61A based on PDB ID: ; 1AYG. To prepare this structure, the distal heme ligand was mutated (M61A) using PYMOL to show the open coordination site for interaction with substrate. (b) CoMC6*a model (ref. 26). The peptide backbones are represented as ribbons, the decapeptide (D) chain in teal, and the tetradeceapeptide (TD) chain in pink. Selected labeled residues are represented as sticks. The amino acid sequence of the D chain is Ac-D-E^2^-U^3^-Q-L-S^6^-U^7^-Q-K-R^10^–NH_2_ and the TD chain is Ac-D-L^2^-Q-Q-L-H^6^-S-Q-K-R^10^-K-I-T-L–NH_2_. (c) Drawing of CoMP11-Ac.

A catalyst that is complexed with a protein or peptide may gain water solubility, stability, and a favorable microenvironment for activity, making engineered metalloprotein catalysts attractive candidates for energy conversion.^1^,^16^,^17^ Metal substitution and mutations in nature's metalloproteins^16^,^18^,^19^ or the incorporation of synthetic catalysts into a protein^20^,^21^ are approaches to engineering novel hydrogenases. For example, *Ht*-CoM61A (Fig. 1a), a cobalt-substituted cytochrome (cyt) *c*_552_ from *Hydrogenobacter thermophilus*, electrocatalytically reduces protons to H_2_ in water at neutral pH with TON > 270 000, but at an overpotential of 730 mV (determined using the half-wave potential as the catalytic potential).^16^ Indeed, engineered proteins for electrocatalytic proton reduction tend to have high overpotentials and/or low TONs.^21^,^22^

Here, we report the H_2_ evolution activity of cobalt mimochrome VI*a (CoMC6*a, Fig. 1b), a synthetic mini-protein containing a cobalt deuteroporphyrin. CoMC6*a is from the mimochrome family, consisting of synthetic miniaturized metalloporphyrin-containing proteins.^23^–^26^ This artificial hydrogenase is based on iron mimochrome VI*a (FeMC6*a), a synthetic peroxidase with enhanced resistance to bleaching.^27^ The CoMC6*a scaffold consists of a distal decapeptide (D) and a proximal tetradecapeptide (TD), each of which is covalently bound to a cobalt deuteroporphyrin through an amide bond between a peptide lysine side chain and a porphyrin propionic acid moiety. The TD “proximal” peptide provides an axial His ligand to the metal ion. CoMC6*a also contains two 2-aminoisobutyric acid (Aib) side chains in the D peptide to enhance helical propensity.^28^–^31^ In this work, we compare the activity of CoMC6*a to CoMP11-Ac, an H_2_-evolution catalyst consisting of a cobalt protoporphyrin IX bound to a single proximal peptide chain (Fig. 1c).^18^ Results indicate that the D peptide chain and mimochrome folding enhance CoMC6*a longevity and lower overpotential relative to CoMP11-Ac.

## Results and discussion

### Characterization of CoMC6*a

The UV-vis spectra of Co(iii)MC6*a (as purified) and Co(ii)MC6*a (prepared by dithionite reduction, Fig. S1, Table S1†) are consistent with a Co(iii)- and Co(ii)-porphyrin species, respectively.^32^ These spectra are very similar to those of CoMC6, which differs from CoMC6*a by three amino acids, two in the D chain and one in the TD chain.^24^ Co(iii)MC6 and Co(ii)MC6 also have been shown to be low-spin with a His axial ligand.^33^ Electrospray ionization-mass spectrometry (ESI-MS) reveals peaks with *m*/*z* values of 1165.32 ([M + 3H]^3+^) and 873.98 ([M + 4H]^4+^), which are consistent with the theoretical value of 3493 Da ([M + H]^+^) (Fig. S2†). Co(iii)MC6*a stock solutions, analyzed for metal contents by atomic absorption spectroscopy, and properly diluted, enabled the calculation of a molar extinction coefficient of (147 000 ± 10 000) L mol^–1^ cm^–1^ for Co(iii)MC6*a at 410 nm and of (213 000 ± 10 000) L mol^–1^ cm^–1^ at 391 nm for Co(ii)MC6*a.

**Fig. 2:**
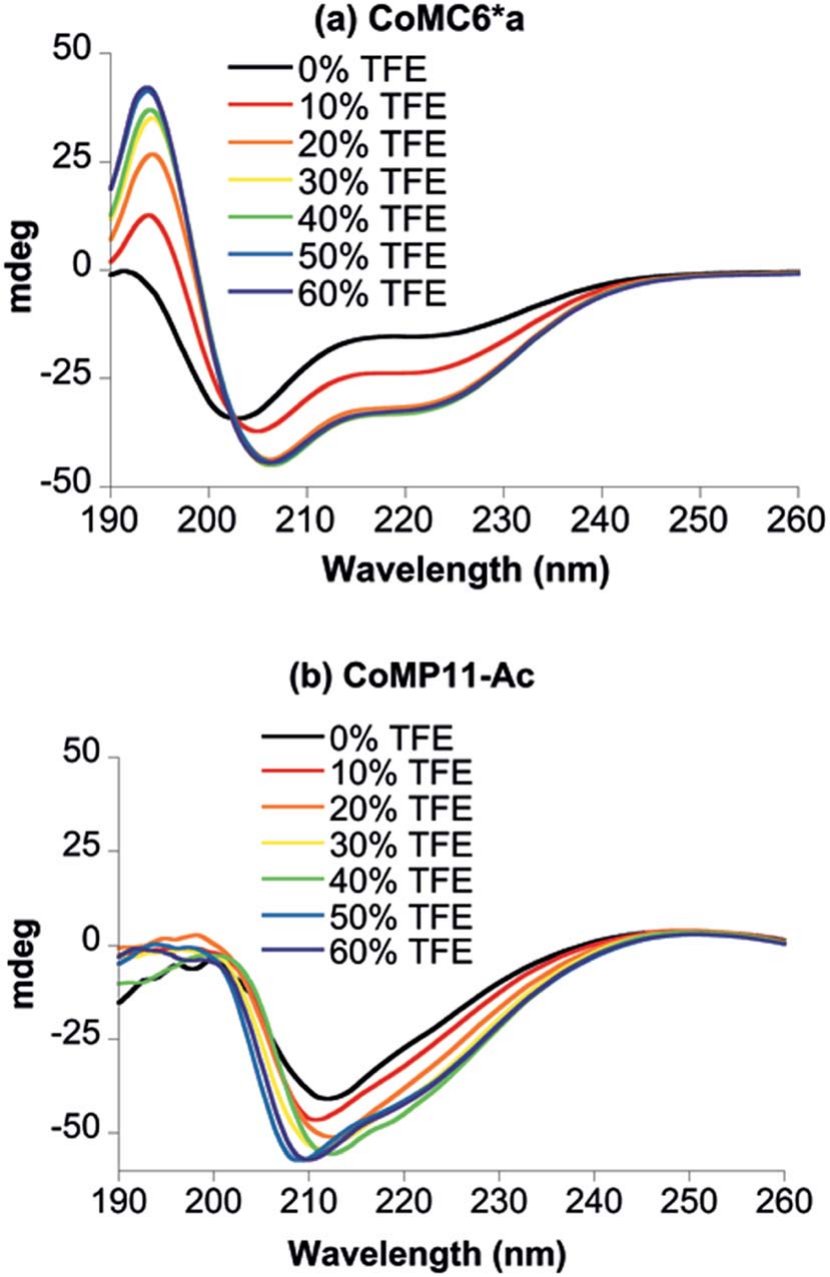
CD spectra of 100 μM CoMC6*a (a) and 100 μM CoMP11-Ac (b) recorded in 10 mM piperazine buffer (pH 6.5) with 0–60% (v/v) TFE.

Circular dichroism (CD) spectra were recorded to assess the secondary structure of Co(iii)MC6*a in the presence of various amounts of trifluoroethanol (TFE), which is known to enhance folding (Fig. 2a).^34^–^37^ All spectra were characterized by negative Cotton effects at 220–222 nm (amide transition, n–π*) and 202–207 nm (amide transition, π–π*, parallel coupling), and a positive Cotton effect at 190 nm (amide transition, π–π*, perpendicular coupling).^38^–^40^ In agreement with previous CD data on mimochromes,^34^ secondary structure is enhanced as the TFE : buffer proportion is increased up to a proportion of 1 : 1 (v/v) TFE : buffer. The isosbestic point at 203 nm indicates that there is a transition between two states under these conditions (Fig. 2a). CD spectra of CoMP11-Ac were also recorded as a function of the proportion of TFE. The spectrum of CoMP11-Ac lacks the peaks that indicate α-helical structure and instead resembles spectra of peptides in a random coil conformation with one negative Cotton effect in the far-UV region at ∼210 nm (Fig. 2b).^41^ Furthermore, the CD spectrum of CoMP11-Ac shows minimal change in response to the change in the proportion of TFE.

### Electrocatalytic H_2_ evolution activity of CoMC6*a in water

**Fig. 3:**
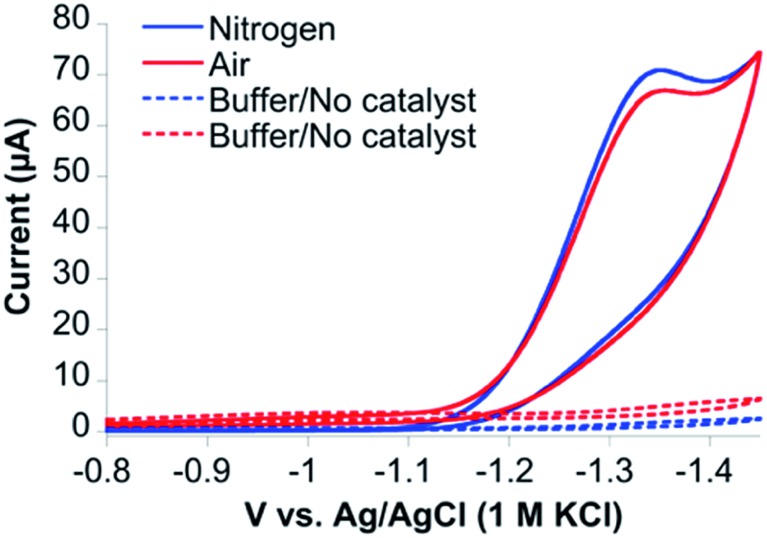
Cyclic voltammograms of CoMC6*a under nitrogen (blue) and air (red) at 100 mV s^–1^. CVs of 1.0 μM CoMC6*a were collected in 100 mM piperazine buffer (pH 6.5) and 100 mM KCl. CVs without catalyst were also collected under nitrogen (dashed blue line) and air (dashed red line). A Pt counter electrode, Ag/AgCl (1 M KCl) reference electrode, and a HMDE working electrode were used.

Cyclic voltammetry (CV) of CoMC6*a in 100 mM piperazine buffer (pH 6.5) and 100 mM KCl under a nitrogen atmosphere showed faradaic current beginning at an onset potential of –1.10 V and a cathodic catalytic wave peak (*E*_p_) at –1.35 V *vs.* Ag/AgCl (1 M KCl), corresponding to a half-wave potential of –1.23 V and a maximum current density of 24.7 A m^–2^ (all potentials herein are reported *vs.* Ag/AgCl (1 M KCl), unless stated otherwise) (Fig. 3). The CV of CoMC6*a under air resulted in similar features in the cyclic voltammogram. The peak current (*i*_p_) was found to be dependent on pH and catalyst concentration, whereas the *E*_p_ is independent of these factors (Fig. S3–S5†). At low catalyst concentrations (below ∼1.5 μM), *i*_p_ increases linearly with the catalyst concentration (Fig. S5†). The linear dependence of *i*_p_ on the square root of the scan rate is consistent with catalyst reduction being diffusion controlled (Fig. S6 and S7†).^42^

To detect the H_2_ product and assess CoMC6*a faradaic efficiency (FE) and longevity, controlled potential electrolysis (CPE) experiments on CoMC6*a were performed. First, short 60 second CPE experiments were run over a range of potentials (–1.15 V to –1.35 V *vs.* Ag/AgCl (1 M KCl)) in 2.0 M piperazine buffer, 0.50 M KCl, pH 6.5 (Fig. S8†). The amount of charge passed increases starting at –1.20 V and reaches a maximum at –1.30 V. This result corresponds to the onset of catalysis at an overpotential of ∼580 mV while the maximum current is obtained at an overpotential of ∼680 mV (eqn (3), Experimental section; Fig. S8 and S9†).

**Fig. 4:**
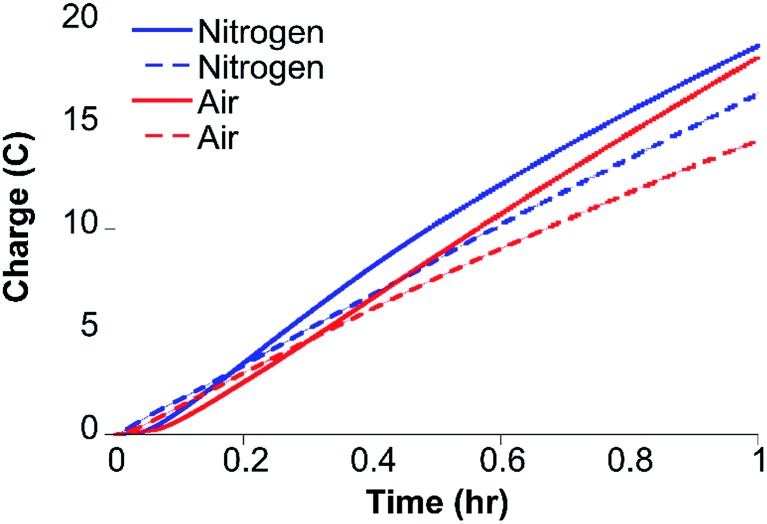
Results of 1 hour CPE at –1.30 V of 0.20 μM CoMC6*a in 2.0 M piperazine (pH 6.5) and 0.50 M KCl in the presence of air (solid red line) and under nitrogen (solid blue line) show that H_2_ evolution by CoMC6*a is minimally impacted by oxygen under these conditions. One-hour CPE experiments under the same conditions under nitrogen (dashed blue line) and air (dashed red line) using the Genesee River as the source of water show that activity in this water source is similar to that in purified water. A Pt counter electrode, a Ag/AgCl (1 M KCl) reference electrode, and a mercury pool working electrode were used.

Gas chromatography (GC) analysis reveals that H_2_ is produced quantitatively during 1 hour CPE (–1.30 V) experiments (Fig. 4) with FE values of 96 ± 2% and 97 ± 1% under nitrogen and air, respectively, showing that H_2_ evolution by CoMC6*a is not impacted by the presence of oxygen under these conditions. UV-vis spectra of Co(ii)MC6*a exposed to air do not indicate the formation of a detectable Co(ii)–O_2_ complex, such as what is seen in cobalt-substituted hemoglobin and myoglobin,^43^ but instead reveal slow oxidation to Co(iii) (Fig. S10†). In addition, the rate of charge build-up and the FE were not significantly affected when unpurified water from the Genesee River was used as solvent, giving FE values of 90 ± 3% and 86 ± 2% under nitrogen and air, respectively (Fig. 4). CPE experiments of 3 hours were performed to test the longevity of the catalyst. The rate of increase in charge passed decreases significantly after 75 minutes (Fig. S11†). The total TON determined after 3 hours is ∼230 000 under nitrogen and ∼220 000 in the presence of oxygen. FE values of 93 ± 2% and 86 ± 3% were determined under nitrogen and air, respectively, suggesting a small amount of oxygen sensitivity in this longer experiment. Other cobalt-based electrocatalysts for hydrogen evolution that are described as oxygen-tolerant have FEs ranging from 43–96% (Table S2†).^14^,^18^,^44^,^45^

There is a short lag time observed in CPE experiments, followed by a nearly linear increase in charge passed for a time period of ∼60 min. Adsorption of catalyst onto the electrode causing an increase of effective catalyst concentration may contribute to the observed lag time, and thus a test for adsorption was performed. After a 30 min CPE experiment with 0.20 μM catalyst in 2.0 M piperazine buffer (pH 6.5), the mercury pool electrode was rinsed with 2.0 M piperazine buffer (pH 6.5) without disturbing the mercury pool, filled with fresh buffer, and then subjected to another 30 min of CPE. A slight current enhancement was observed compared to the background, at a level that is ∼10% of that obtained with 0.2 μM catalyst (Fig. S12†). We suggest that the lag time for the CPE experiments corresponds to the slow reduction of Co(iii) to Co(ii).

**Fig. 5:**
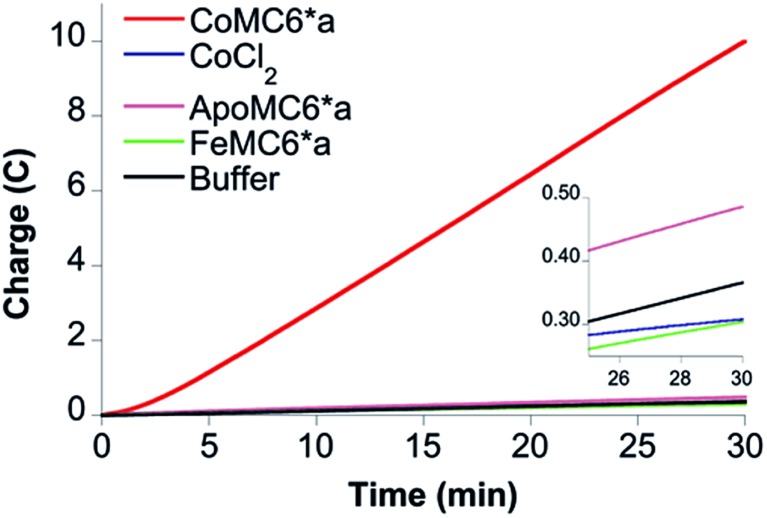
CPE control experiments. Results of 30 min CPE experiments on 0.20 μM CoMC6*a (red), ApoMC6*a (brown), FeMC6*a (green), CoCl_2_ (purple) in 2.0 M piperazine (pH 6.5) and 0.50 M KCl at an Hg pool working electrode. The inset shows the results on the control samples with an expanded vertical scale.

To verify that CoMC6*a is the active catalyst, CPE experiments were performed with CoCl_2_ and MC6*a analogs (ApoMC6*a and FeMC6*a), none of which yielded significant passed charge (Fig. 5). This result indicates that CoMC6*a with cobalt complexed by the porphyrin is responsible for the observed activity.

### Electrocatalytic H_2_ evolution catalyzed by CoMC6*a as a function of % TFE in water

**Fig. 6:**
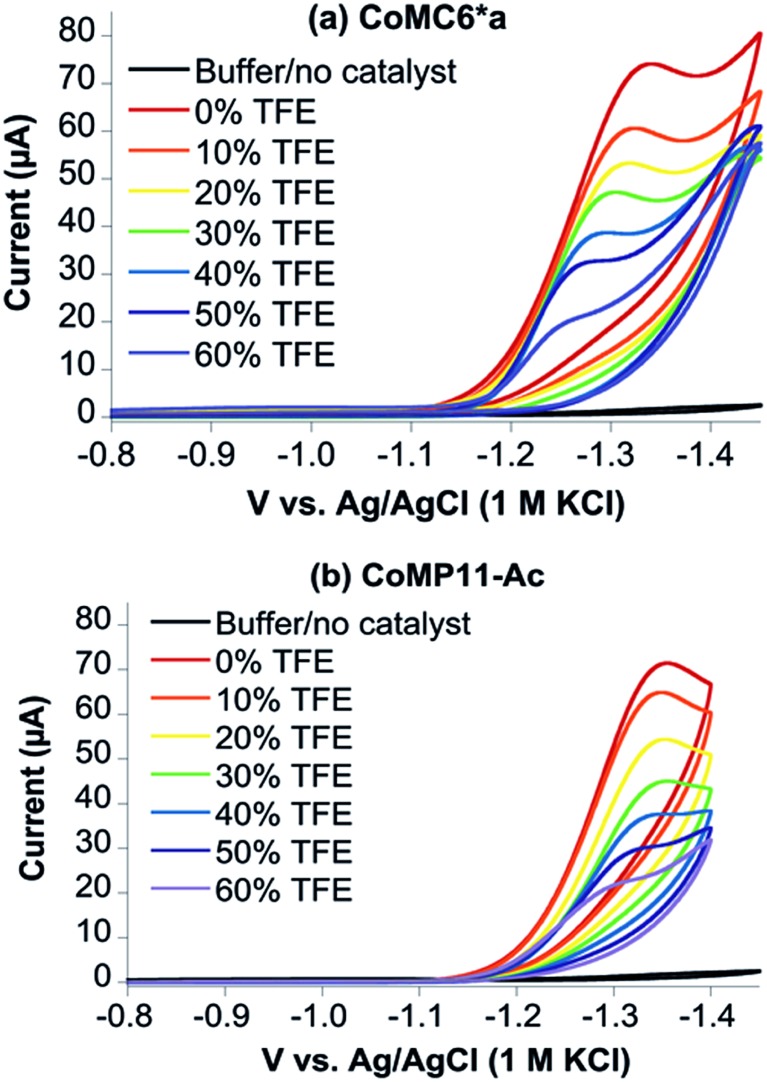
Cyclic voltammograms of CoMC6*a (a) and CoMP11-Ac (b) as a function of % TFE. CVs of 1.0 μM CoMC6*a (a) and 1.0 μM CoMP11-Ac (b) with increasing proportions of TFE were recorded in 100 mM piperazine buffer (pH 6.5) and 100 mM KCl. The scan rate is 100 mV s^–1^ and an HMDE is the working electrode.

**Fig. 7:**
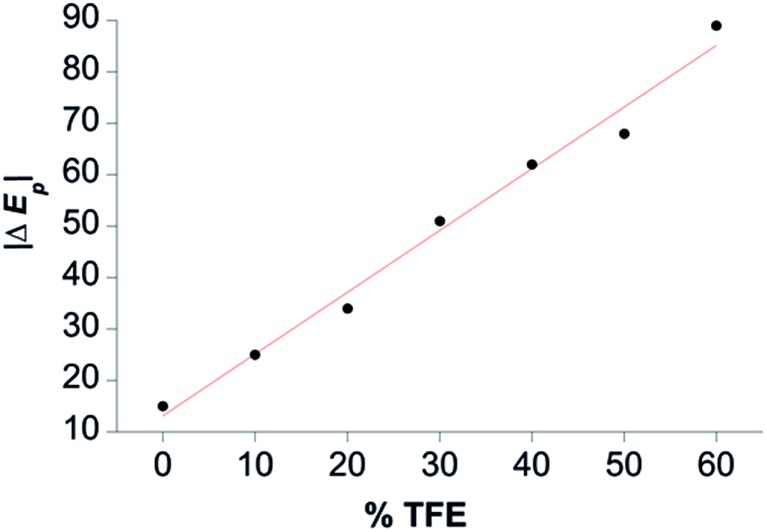
Difference between CoMC6*a and CoMP11-Ac catalytic peak potentials as a function of % TFE (Δ*E*_p_). The magnitude of Δ*E*_p_ is plotted as a function of % TFE, and the red line shows a linear fit to the data. See Fig. S13† for *E*_p_ values.

TFE increases the helicity of peptide chains, contributing to the overall folding of a protein or peptide.^34^ Further characterization of CoMC6*a as an electrocatalyst for proton reduction as a function of its TFE-induced folding was carried out by CV (Fig. 6). As a reference, CVs were collected under the same conditions on CoMP11-Ac, a cobalt porphyrin-peptide H_2_-evolution catalyst with a single axial His-donating peptide that does not undergo significant TFE-induced folding (Fig. 2). Both experiments were performed using 1.0 μM catalyst, 100 mM piperazine buffer (pH 6.5) and 100 mM KCl. TFE concentrations were varied from 0% to 60% in increments of 10%. The pH of these solutions deviates only slightly, from 6.5 in 0% TFE to 6.4 in 60% TFE. As seen in Fig. 6, both catalysts show a similar decrease in peak current with increasing proportion of TFE. We suggest that this change in peak current results from the effect of TFE on biocatalyst solvation. Water/TFE solutions experience a decrease in solution free energy relative to water, which raises the entropic cost of moving bulk water molecules to the protein's solvation layer. This effect then would decrease the availability of protons near the catalyst, and/or transfer of protons from solution to the catalyst.^35^,^46^ CoMC6*a also shows a 100 mV anodic shift in the peak potential from –1.34 V to –1.24 V with increasing TFE proportion. In contrast, CoMP11-Ac shows minimal change in peak potential with TFE proportion (Fig. S13†). To consider the specific effect of CoMC6*a folding on overpotential, CoMP11-Ac was used as a reference by plotting the difference between peak potentials for CoMC6*a and CoMP11-Ac as a function of % TFE (Fig. 7). This plot gives a clear linear trend of the difference in catalytic peak potentials (Δ*E*_p_) as a function of % TFE and suggests a 90 mV decrease in overpotential for CoMC6*a relative to CoMP11-Ac attributed to TFE-induced folding of CoMC6*a.

While CoMP11-Ac yields a high current density of 208 A m^–2^, its longevity is poor, and it shows significant degradation after ∼20 minutes of CPE at –1.35 V.^18^ Here, we show that a cobalt deuteroporphyrin sandwiched by proximal and distal peptides displays significantly enhanced longevity, with activity lasting for hours rather than minutes. As a result, CoMC6*a has a high TON ∼ 230 000, which is a nine-fold improvement over CoMP11-Ac (TON ∼ 25 000).^18^ A similarly enhanced stability and high TON for H_2_ evolution was also observed for the M61A mutant of cobalt-substituted *H. thermophilus* cytochrome *c*_552_ (*Ht*-CoM61A) (Fig. 1a).^16^ This longevity was attributed to *Ht*-CoM61A being a thermophilic 80-amino acid protein with a buried active site. Thus, the similar robustness of CoMC6*a was unexpected. Another important difference between *Ht*-CoM61A and CoMC6*a is that *Ht*-CoM61A was found to adsorb significantly to the electrode, retaining ∼35% of activity in a rinse test,^16^ whereas rinsing the electrode exposed to CoMC6*a yields ∼10% residual activity. We propose that the greater adsorption of *Ht*-CoM61A results from the higher hydrophobicity of its polypeptide relative to CoMC6*a.

Overpotential is used as a metric for evaluating the efficiency of an electrocatalyst, and there have been different approaches to lowering catalyst overpotential. One approach is inspired by the ability of hydrogenases to shuttle protons directly to the active site.^47^ In [FeFe]-hydrogenase, theoretical, spectroscopic and biochemical studies indicate that a 2-aza-1,3-dithiolate bridge to the two iron ions in the H-cluster is responsible for relaying protons to the iron center *via* the amine.^48^,^49^ In the case of [NiFe]-hydrogenases, protonation of the thiol of a Cys ligand to nickel is invoked in the proton transfer pathway.^4^,^50^,^51^ There has been success in installing proton relays in the secondary coordination sphere of catalysts,^16^ such as the pendant amine in Dubois-type [Ni(PR2NR′2)_2_]^2+^ catalysts and the pendant acid relay in “hangman” nickel or cobalt porphyrins.^9^,^52^ In the case of the cobalt- or nickel-dithiolene complexes, the sulfur donor to the metal is protonated, which is analogous to the proposed protonation of the Cys ligand in [NiFe]-hydrogenases. Here, for CoMC6*a, we observe that enhanced peptide folding lowers the overpotential by ∼90 mV relative to CoMP11-Ac. While the basis for this observation is not yet understood, we noted that CoMC6*a folding positions a serine residue (Ser6) on the distal (D) chain of CoMC6*a to possibly assist with shuttling protons to the active site, perhaps by positioning a water molecule. However, a similar shift in overpotential of ∼100 mV was observed in CVs in 0% to 60% TFE for the CoMC6*a variant in which Ser6 in the D chain is replaced with Gly (Ser6Gly) (Fig. S14†). This result indicates that any interactions involving the Ser6 side chain are not responsible for the lowered overpotential in folded CoMC6*a. We also note that Ser6Gly has modestly different CD features compared to CoMC6*a. However, negative Cotton effects at 205–207 nm and 220–222 nm are observed with increasing proportion of TFE, with the most organized secondary structure for Ser6Gly observed at and beyond 40% TFE (v/v) (Fig. S15†). An alternative hypothesis regarding the lowered overpotential in folded CoMC6*a is that peptide folding raises the Co(ii/i) potential. Presuming that a Co(i) species plays a role in catalysis (see next section), this shift may lower overpotential. Additional studies of this phenomenon using a range of mimochrome variants will be pursued.

Complexing an otherwise insoluble catalyst to a peptide or protein scaffold may enable the catalyst to operate in water. Catalysts that gain the ability to function in water, however, often show reduced activity in terms of TON for H_2_ evolution.^19^,^21^ Here, we find that the minimal peptide scaffold in CoMC6*a enables the cobalt porphyrin catalytic moiety to operate in water and provide a high TON. Furthermore, enhanced folding of the α-helical peptides in CoMC6*a by TFE lowers the overpotential by up to 100 mV. The solution-dependent folding of CoMC6*a thus enables the investigation of peptide folding on overpotential.

### Electrocatalysis in aprotic solvent

CoMC6*a was designed to fold in aqueous solution. However, working in aprotic solvent provides the opportunity to control the addition of protons to the catalyst. Thus, electrochemical studies were performed in dimethylformamide (DMF) with 100 mM NBu_4_PF_6_ electrolyte to probe the reactivity of CoMC6*a with protons and/or oxygen. The primary motivation for these studies is to determine whether CoMC6*a catalyzes oxygen reduction under conditions of low or zero proton concentration. Furthermore, we were motivated to observe the Co(iii/ii) and Co(ii/i) redox couples, which were not detected in water. We attribute the lack of an observable Co(iii/ii) couple in water to the expected high reorganization energy of this couple in a highly polar solvent,^53^ while the Co(ii/i) couple is not observed in water as a result of the expected role of Co(i) in proton reduction catalysis.^54^

**Fig. 8:**
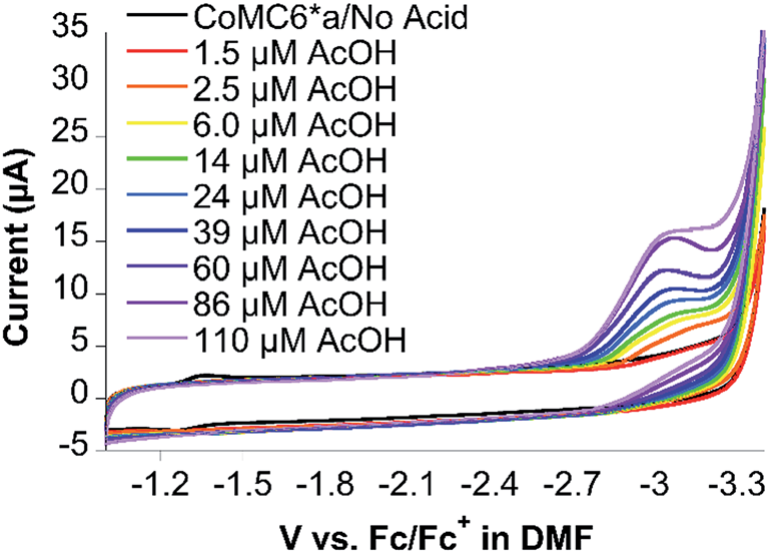
Cyclic voltammograms of CoMC6*a (10 μM) in DMF and 100 mM NBu_4_PF_6_ as a function of added AcOH, where the current increases as the amount of AcOH increases. A glassy carbon working electrode, Pt wire counter electrode, and an Ag wire pseudoreference electrode were used. Ferrocene was used as an internal standard, and the scan rate was 100 mV s^–1^.

CVs of CoMC6*a collected in DMF under nitrogen show quasi-reversible redox transitions at –1.49 V and –2.64 V *vs.* ferrocene/ferrocenium (Fc/Fc^+^), assigned to the Co(iii/ii) and Co(ii/i) couples, respectively (Fig. S16†). Observation of these couples required adjustments to the CV scan rate, with a lower scan rate used to observe the Co(iii/ii) couple due to its expected high reorganization energy leading to slow electron transfer.^53^ Indeed, the Co(iii/ii) couple was not observed by CV in water, which is attributed to the expected higher reorganization energy in water compared to that in DMF.^53^ CVs of CoMC6*a in DMF in the presence of oxygen show similar redox transitions to those observed under nitrogen, indicating that there is no reaction of CoMC6*a with oxygen seen under these conditions (Fig. S17†). CVs of 10 μM CoMC6*a were also collected in DMF and variable (0 to 110 μM) acetic acid (AcOH) under nitrogen and under air. Upon addition of AcOH, faradaic current beginning at an onset potential of ∼–2.60 V *vs.* Fc/Fc^+^, *i.e.*, close to the Co(ii/i) couple, is observed, reaching a cathodic wave peak at –3.0 V *vs.* Fc/Fc^+^ (Fig. 8). The same experiments were attempted in the presence of air, but the glassy carbon electrode in DMF reduces oxygen, observed as a quasi-reversible wave at ∼–1.35 V *vs.* Fc/Fc^+^ (Fig. S18†). Nevertheless, the CV of CoMC6*a in DMF in the presence of air does not yield a catalytic wave relative to background, indicating no catalytic oxygen reduction under these conditions (Fig. S19†). In case protons are needed in any oxygen reduction reaction, additional experiments in which AcOH was added to a DMF solution exposed to air with and without CoMC6*a were collected. These also show no significant enhancement of current by the presence of CoMC6*a (Fig. S20†). The results of these studies in DMF support the hypothesis that a Co(i) species is necessary for H_2_ evolution catalysis by CoMC6*a. Furthermore, they indicate that, under these experimental conditions, catalytic reduction of oxygen by CoMC6*a is not observed. The basis for this lack of reactivity with oxygen is not clear, but a possible reason is that the distal peptide is positioned to destabilize any cobalt–O_2_ adduct. However, H_2_ evolution catalyzed by CoMP11-Ac also is minimally impacted by the presence of oxygen, and this catalyst does not have a distal peptide. Future work will include detailed studies on reactions of these catalysts with oxygen and protons. Analogous data on CoMP11-Ac could not be collected as a result of its insolubility in DMF and other inert organic solvents.

## Conclusions

In summary, we find that CoMC6*a, consisting of two peptides sandwiching a cobalt deuteroporphyrin, displays lower overpotential and enhanced longevity relative to CoMP11-Ac, which has only a single peptide on the “proximal” side of the porphyrin. Furthermore, CoMC6*a reduces protons in the presence of oxygen. This result is a step toward the production of more stable and efficient oxygen-tolerant engineered biocatalysts for H_2_ evolution.

## Experimental section

### Synthesis and characterization CoMC6*a

#### RP-HPLC purification of CoMC6*a

Apo-mimochrome VI*a (ApoMC6*a) and CoMP11-Ac were prepared as previously described.^18^,^27^ ApoMC6*a is the non-metallated version of CoMC6*a. Cobalt insertion into ApoMC6*a was carried out using a modified version of the acetic acid/acetate method.^27^,^55^ Purified ApoMC6*a (100 mg) was dissolved in 60 : 40 AcOH : TFE (v/v) at a concentration of 20 mg mL^–1^, along with a 20-fold molar excess of cobalt(ii) acetate. The reaction was performed under a nitrogen atmosphere at 50 °C for 2 hours. Metal insertion was monitored by analytical RP-HPLC (Shimadzu LC-10ADVP equipped with a SPDM10AVP diode-array detector and Vydac C18, 150 mm × 4.6 mm column) with an elution gradient of 50% to 80% acetonitrile, 0.1% trifluoroacetic acid (TFA) over 35 min with a flow rate of 1 mL min^–1^, monitored by UV-vis spectroscopy (Varian Cary 50) at 410 nm. The reaction products were concentrated under vacuum, re-dissolved in 10 mL water, 0.1% TFA (v/v) and desalted by flash chromatography performed with a Biotage Isolera flash purification system, equipped with a diode-array detector. CoMC6*a was loaded on a 30 g SiO_2_ C18 reversed-phase column with an elution gradient from 0 to 95% acetonitrile, 0.1% TFA over 20 min with a flow rate of 25 mL min^–1^. Similar fractions were pooled and concentrated. Lyophilization yielded the final product (95% purity).

#### ESI-MS characterization of CoMC6*a

Lyophilized samples from the purification step were characterized by ESI-MS (Shimadzu LCMS-1010EV system with ESI interface, Q-array-octupole-quadrupole mass analyzer, and Shimadzu LC-MS Solution Workstation software for data processing). The optimized MS parameters were selected as follows: curved desolvation line (CDL), 250 °C; block temperature, 250 °C; probe temperature, 250 °C; detector gain, 1.6 kV; probe voltage, +kV; CDL voltage, –15 V.

#### UV-vis spectroscopy

UV-vis (Varian Cary 50 equipped with a temperature controller) absorption spectra during the synthesis and purification steps were measured at 25 °C from 200–800 nm with a scan rate of 200 nm min^–1^, a 2.0 nm data interval and an averaging time of 0.0125 s. Alternatively, spectra were collected on a Shimadzu 8452 UV-vis spectrophotometer in the range of 200–800 nm with a scan rate of 250 nm min^–1^, 1.0 nm data interval, and 1.0 nm slit width.

#### Atomic absorption spectroscopy

Atomic absorption spectroscopy was used for determining the CoMC6*a concentrations on the basis of metal content. A stock solution of approximately 10^–4^ M Co(iii)MC6*a in ultra-pure metal-free water, 0.1% TFA was prepared. Three aliquots from the stock solution (50 μL each) were treated with 200 μL of 69% ultra-pure nitric acid for 2 hours at 90 °C. Mineralized samples were diluted with ultra-pure water to achieve a cobalt concentration of ∼10 μg L^–1^ (ppb). The cobalt concentration in the samples was read by using a Shimadzu AA700 atomic absorption spectrophotometer, equipped with the autosampler ACS-7000 and the graphite furnace atomizer GFA-7000. Co(iii)MC6*a stock solutions, analyzed for metal content, were appropriately diluted and used for determining the extinction coefficients at the Soret band maximum wavelength (410 nm), by plotting the absorbance as a function of concentration.

#### Circular dichroism

CD (Jasco J-1100) spectra were collected to assess the secondary structure of the catalysts in the far-UV region (190–260 nm). The spectra were collected at 0.1 nm intervals with a 100 nm min^–1^ scan rate, 1.0 nm bandwidth and a 4 s response. Five accumulations were collected for each measurement. Spectra are reported in millidegrees (mdeg). Experiments were run at 25 °C in 10 mM piperazine buffer (pH 6.5) with 0% to 60% (v/v) trifluoroethanol (TFE) in increments of 10%.

### Electrochemistry

#### Controlled potential electrolysis

Electrochemical experiments were carried out with a 620D potentiostat (CH instruments). CPE was performed in a custom-built electrolysis cell (Adams & Crittenden) with three equi-volume chambers of 1.5 cm diameter and 6 cm height. The chambers are separated by 10 mm diameter P5 (1.0–1.6 μm pore size) glass. The central cell compartment contains a mercury pool electrode with a surface area of 1.6 cm^2^, which is connected to the potentiostat through an insulated platinum wire, and each compartment is sealed with a gas-tight septum. A coiled platinum electrode with a surface area of 2.3 cm^2^ served as the counter electrode and an Ag/AgCl (1 M KCl) electrode (CH Instruments, Inc.) served as the reference electrode. Mercury was chosen as a working electrode for electrochemical experiments performed in water because of its large cathodic window and low background. In addition, mercury forms an amalgam with cobalt, inhibiting catalysis by any nanoparticles that may form at the electrode.^56^–^58^ Before the start of each experiment, the mercury was cleaned using 0.10 M NaOH in water by applying –1.8 V *vs.* Ag/AgCl (1 M KCl) for 30 min. CPE studies were performed using 5 mL of 0.20 μM CoMC6*a in 2.0 M piperazine buffer (pH 6.5) and 0.50 M KCl. CPEs that exceeded one hour used 50 nM of CoMC6*a to prevent excess build-up of pressure in the CPE cell. The Genesee River water was collected from the river bank near Hutchison Hall, University of Rochester, and filtered through a 0.20 μm membrane to separate particulates and buffered with 2.0 M piperazine (pH 6.5).

Prior to each experiment, the CPE cell was purged for 15 minutes with a N_2_/CH_4_ gas mixture (80/20) to allow use of CH_4_ as an internal standard. H_2_ quantification was performed by GC (Shimadzu GC-2014, thermal conductivity detector, Rt-Msieve 5A column) analysis of cell head space samples. For the experiments under nitrogen, the ratio of H_2_/CH_4_ peak areas was compared to a standard calibration curve. For experiments under air, the H_2_ peak area was compared to a calibration curve that was made by injecting known amounts of H_2_ into a sealed CPE cell containing house air. Once the amount of H_2_ evolved was measured, turnover number (TON) and faradaic efficiency (FE) were calculated using the following equations, where 235 mV is the conversion from the Ag/AgCl (1 M KCl) reference electrode to the SHE reference electrode, and 385 mV is the oxidation potential *vs.* SHE of the H^+^/H_2_ couple at pH 6.5:

1 FE (%) = (*n*e × *F*)/*QT* × 100%

2 TON = *n*H_2_/*n*CoMC6*a

3 Overpotential (in CPE) = |applied potential + 235 mV + 385 mV|

4 Overpotential (in CV) = |half-wave potential + 235 mV + 385 mV|

#### Cyclic voltammetry in water

Unless indicated otherwise, CV experiments in water were performed with a hanging mercury drop electrode (HMDE) from BASi Instruments. The general procedure for CV is as follows: 5.0 mL of 1.0 μM CoMC6*a in 100 mM KCl and 100 mM piperazine buffer (pH 6.5) was used. CV scan rates were 100 mV s^–1^ unless stated otherwise. Each measurement was repeated twice and a fresh mercury drop was used for each experiment. Before each experiment, the solution was purged for 15 min with nitrogen. The measurements were performed under an atmosphere of nitrogen unless specified otherwise. To perform pH titrations, the pH was adjusted by adding 10 μL of 1.0 M HCl or 1.0 M NaOH at a time. The pH was monitored using a VWR SB70P pH meter and a Mettler Toledo InLab semimicro pH probe.

#### Cyclic voltammetry in DMF

DMF was dried overnight over 4 Å molecular sieves. The molecular sieves were first activated by flaming and then placing under vacuum for 20 minutes; these steps were carried out in a 250 mL round-bottom flask. After the sieves cooled to room temperature, DMF was added to the flask, purged with argon, and stored in a Vac Atmospheres glove box under nitrogen. The CVs in DMF were performed in the glove box, with the O_2_ content < 8 ppm. CVs in DMF were performed with a glassy carbon electrode as the working electrode, a coiled Pt wire as the counter electrode, and an Ag wire as the pseudoreference electrode. The general procedure is as follows unless stated otherwise: 5 mL of 10 μM CoMC6*a in DMF and 100 mM NBu_4_PF_6_ were used. The measurements were performed under one atmosphere of nitrogen unless stated otherwise. Ferrocene was included as an internal reference, and potentials are reported by taking the Fc/Fc^+^ couple as 0 V in order to report the potentials *vs.* Fc/Fc^+^.

## Conflicts of interest

There are no conflicts of interest to declare.

## Supplementary Material

Supplementary information
