# Supplementary material for: Hydrogen evolution from water catalyzed by cobalt-mimochrome VI*a, a synthetic mini-protein
Source: Chem Sci. 2018 Sep 14;9(45):8582–9. doi: 10.1039/c8sc01948g (PMC6253682; doi:10.1039/c8sc01948g)
Supplement: Supplementary file 1 [file SC-009-C8SC01948G-s001.pdf]

Electronic Supplementary Material (ESI) for Chemical Science.
This journal is © The Royal Society of Chemistry 2018

Supporting Information

Hydrogen evolution from water catalyzed by cobalt-mimochrome VI*a, a synthetic mini-protein

Vincenzo Firpo, Jennifer M. Le, Vincenzo Pavone, Angela Lombardi,* and Kara L. Bren*

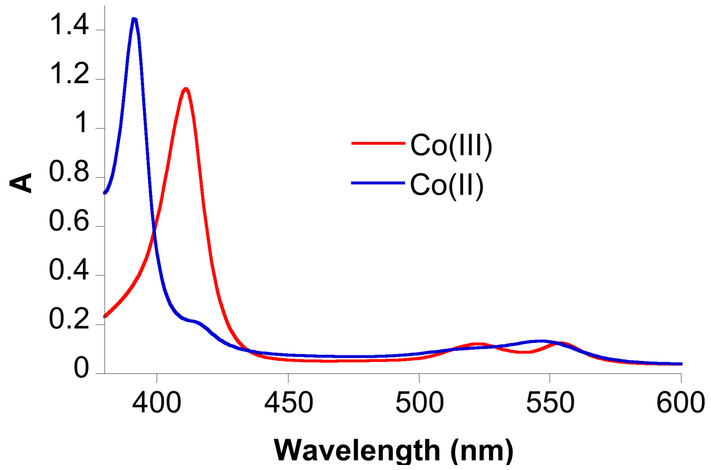

Fig. S1 Absorption spectra of Co(III)MC6*a (red) and Co(II)MC6*a (blue), indicative of Co(II) and Co(III)-porphyrins, respecitively.[1] The spectrum of Co(II)MC6*a was taken after the addition of 50-molar excess of dithionite. Samples are in water and under nitrogen atmosphere.

Table S1. Soret and Q-bands of Co(III)MC6*a and Co(II)MC6*a.

	Soret (nm)	Q-bands (nm)	Soret extinction coefficient
**Co(III)MC6*a**	410	554, 523	147,000 ± 10,000 $L \cdot mol^{-1} \cdot cm^{-1}$
**Co(II)MC6*a**	391	547	213,000 ± 10,000 $L \cdot mol^{-1} \cdot cm^{-1}$

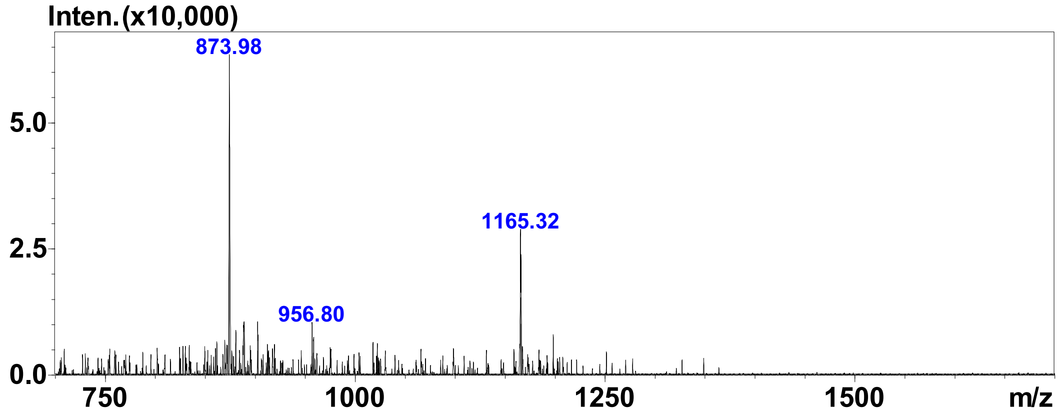

Fig. S2 ESI-MS spectrum of Co(III)MC6*a. The peaks at *m/z* 1165.32 [$M+3H^+$] and 873.98 [$M+4H^+$] are consistent with theoretical value of 3493 Da ([$M+H]^+$).

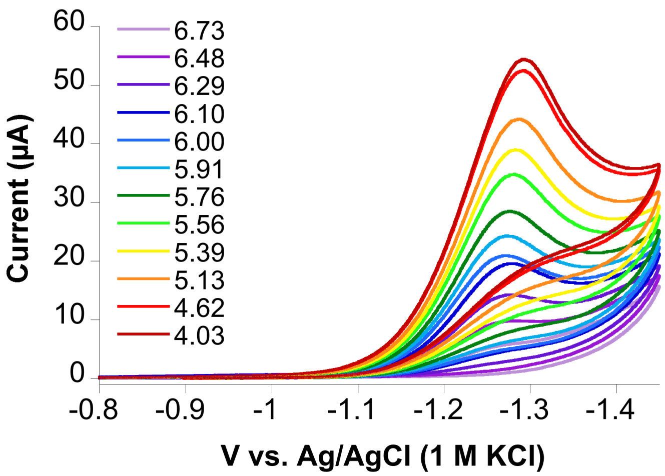

Fig. S3 Effect of pH on proton reduction activity. CVs are of 1.0 μM CoMC6*a in 10 mM piperazine and 100 mM KCl as a function of pH at 100 mV/s scan rate, using a hanging mercury drop electrode (HMDE). The current increases as pH decreases.

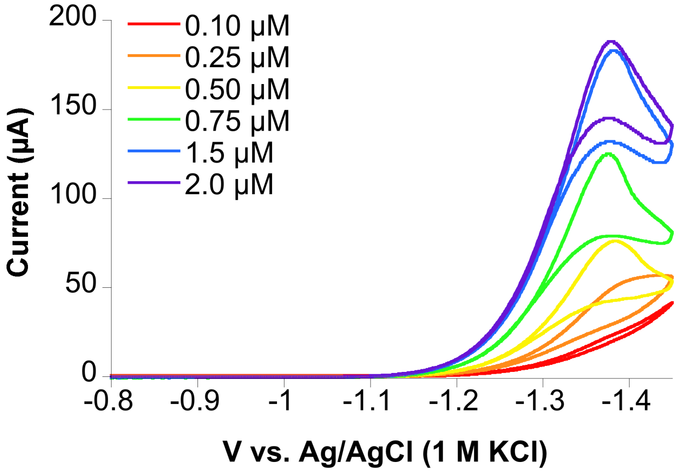

Fig. S4 Effect of catalyst concentration on proton reduction activity. CVs of CoMC6*a collected at varying catalyst concentrations (0.10 μM – 2.0 μM, red to violet traces). Samples were in 2.0 M piperazine, 0.50 M KCl, pH 6.5. A scan rate of 500 mV/s was used to induce catalyst-limiting conditions.

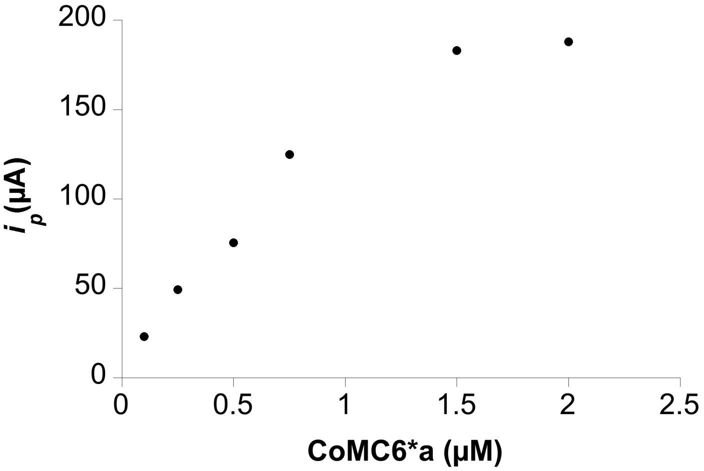

Fig. S5 Plot of peak current (i_p) vs. CoMC6*a concentration (for the data in Fig. S4).

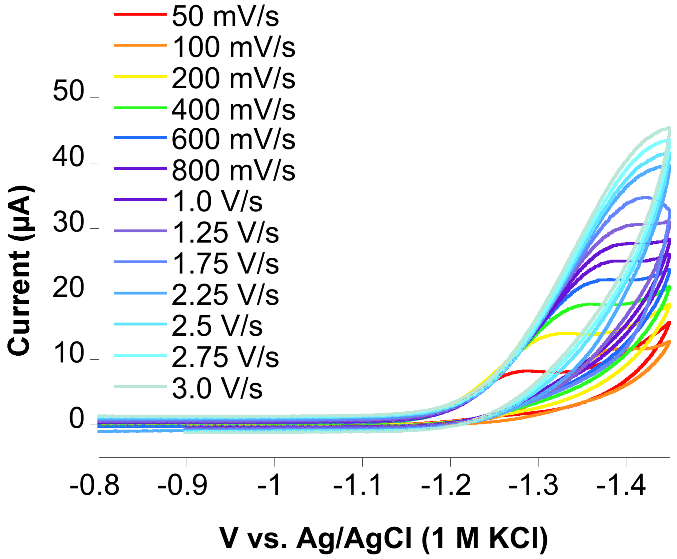

Fig. S6 Cyclic voltammograms of 1.0 μM CoMC6*a in 100 mM piperazine (pH 6.5), 100 mM KCl as a function of scan rate (50 mV/s, red, up to 3.0 V/s, light blue).

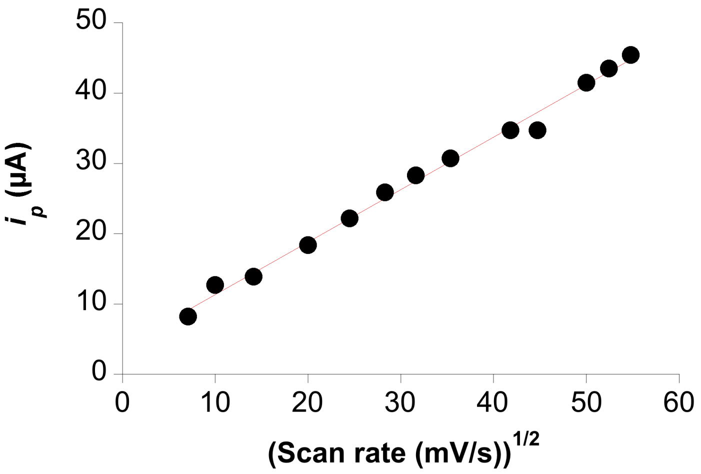

Fig. S7 Plot of i_p vs. square root of scan rate for the data in Fig. S6.

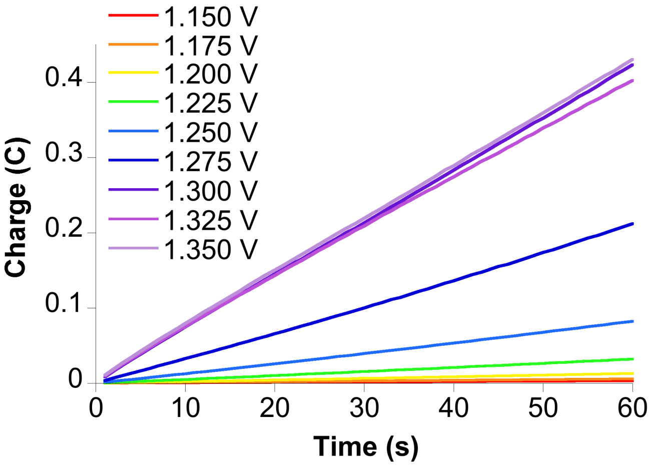

Fig. S8 Charge passed in 60-second CPE experiments at an Hg pool electrode as a function of applied potential ranging from −1.15 V to −1.35 V vs. Ag/AgCl (1 M KCl). The sample is 1.0 μM CoMC6*a in 2.0 M piperazine (pH 6.5), 0.50 M KCl.

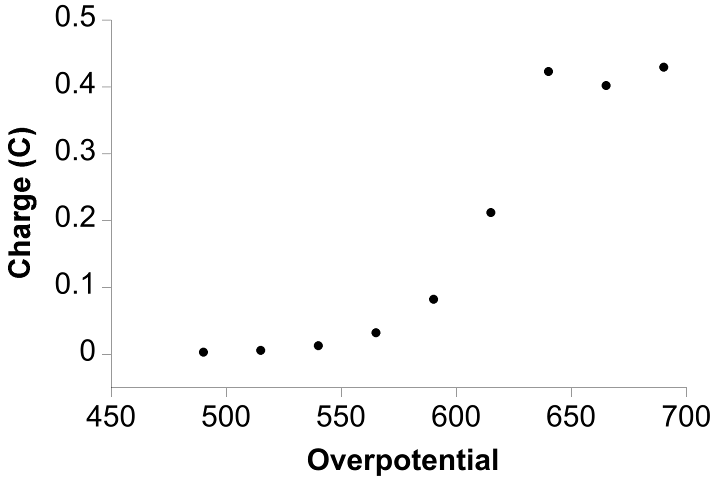

Fig. S9 Plot of charge passed as a function of overpotential (Equation 3, Experimental Section) in 60-second CPE experiments (Fig. S8) on 1.0 μM CoMC6*a in 2.0 M piperazine (pH 6.5) and 0.50 M KCl at an Hg pool electrode.

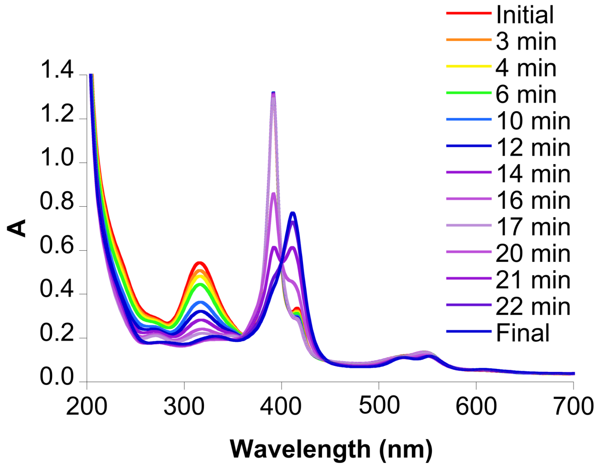

Fig. S10 UV-vis spectra of Co(II)MC6*a exposed to oxygen as a function of time (0 minutes, red, to 22 minutes, violet). Co(III)MC6*a was reduced to Co(II)MC6*a with a 50-fold molar excess of sodium dithionite in water, and then exposed to air. The Soret band λ_{max} at 391 nm is the same as that observed for reduced Co(II)MC6*a prepared and measured under nitrogen (Fig. S1, Table S1). After 22 minutes, the Co(II)MC6*a is oxidized to Co(III)MC6*a, with a Soret band λ_{max} at 410 nm. The peak at 316 nm is from dithionite.

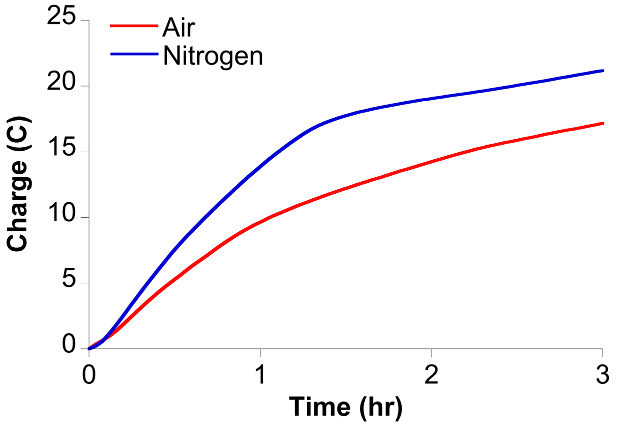

Fig. S11 Controlled potential electrolysis (–1.30 V vs. Ag/AgCl (1 M KCl)) of 50 nM CoMC6*a at an Hg pool electrode under nitrogen (blue line) and air (red line). This experiment was carried out in 2.0 M piperazine (pH 6.5) and 0.50 M KCl. Comparison of charge passed to H_2 produced indicates faradaic efficiencies of 86% (air) and 93% (nitrogen).

Table S2. Comparison of oxygen-tolerant H_2-evolution electrocatalysts in water.

Catalyst	TON	FE	Reference
**CoMC6*a (N_2)**	230,000	92%	This work
**CoMC6*a (O_2)**	220,000	86%	This work
**CoMP11-Ac (N_2)**[2]	25,000	98%	2
**CoMP11-Ac (O_2)**[2]	19,000	85%	2
	270,000	96%	3
**CoGGH (N_2)**[3]	310	92%	4
**CoGGH (O_2)**[3]	450	88%	4
**CoP^{-} (N_2)**[4]	__	68%	5
**CoP^{-} (O_2)**[4]	__	43%	5
**Co-F8 (N_2)**[5]	10,000,000	__	6
**Co-F8 (O_2)**[5]	10,000,000	52%	6

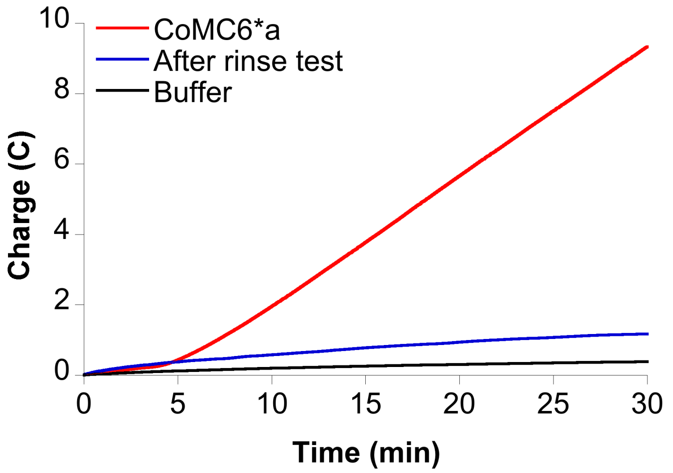

Fig. S12 Controlled potential electrolysis (–1.30 V vs. Ag/AgCl (1 M KCl)) of 0.2 μM CoMC6*a in 2.0 M piperazine (pH 6.5) (red) and of 2.0 M piperazine (pH 6.5) after rinsing the electrode (blue). The black trace is the background from the buffer.

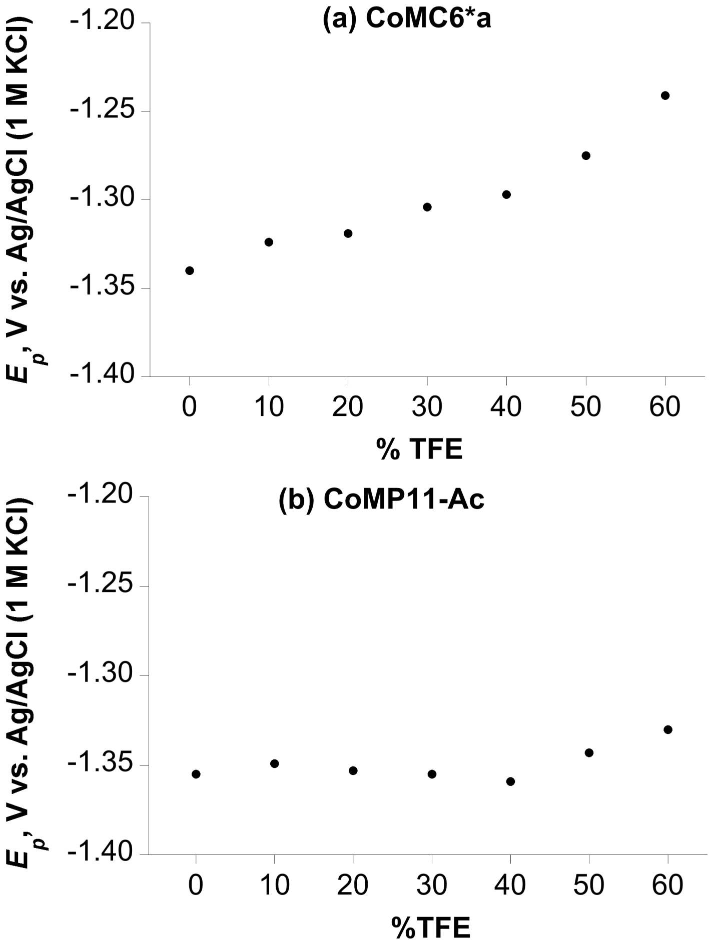

Fig. S13 E_p as a function of %TFE from CV of 1.0 μM CoMC6*a (a) and 1.0 μM CoMP11-Ac (b) in 100 mM piperazine (pH 6.5) and 100 mM KCl.

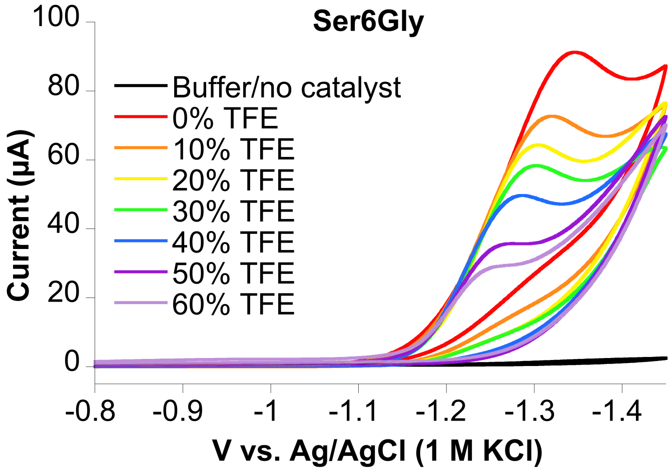

Fig. S14 Cyclic voltammograms of 1.0 µM Ser6Gly CoMC6*a with increasing proportions of TFE recorded in 100 mM piperazine buffer (pH 6.5) and 100 mM KCl. There is a 100-mV shift in peak potential from –1.33 to –1.23 V vs. Ag/AgCl (1 M KCl) from 0% to 60% TFE. The scan rate is 100 mV/s.

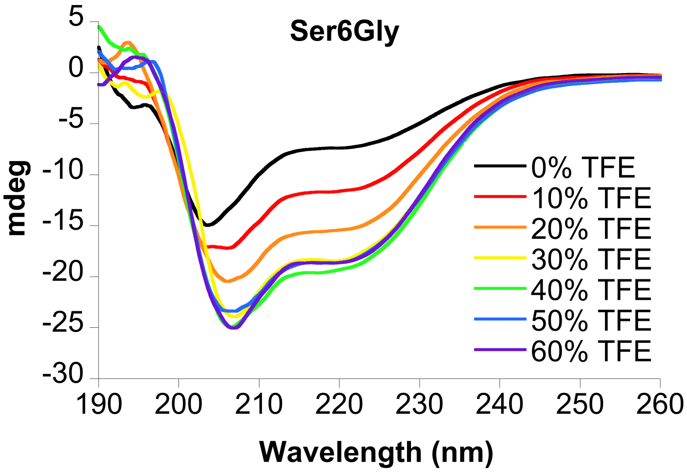

Fig. S15 Circular dichroism spectra of 100 µM Ser6Gly CoMC6*a recorded in 10 mM piperazine buffer (pH 6.5) with 0 – 60% (v/v) TFE. Negative Cotton effects at ~206 nm and ~222 nm are observed with increasing proportion of TFE.

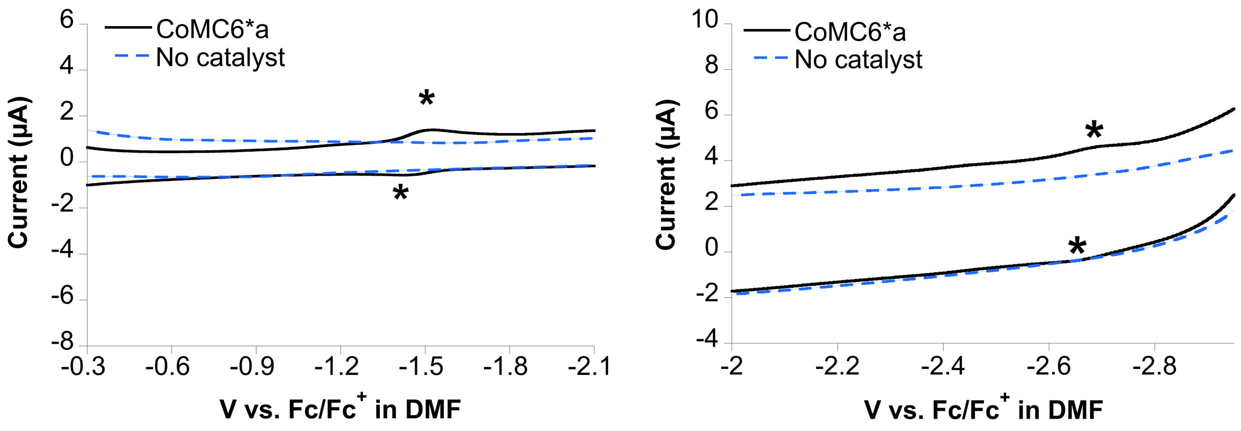

Fig. S16 The Co(III/II) and Co(II/I) redox couples observed by CV on 100 μM CoMC6*a nitrogen in DMF with 100 mM NBu_4PF_6 electrolyte. **Left panel:** The Co(III/II) (~–1.49 V vs. Fc/Fc^+) couple resolved at a 50 mV/s scan rate. **Right panel:** The Co(II/I) (~–2.64 V vs. Fc/Fc^+) couple resolved at a 500 mV/s scan rate. Peaks are marked with an asterisk (*). These experiments used a glassy carbon working electrode, a Pt counterelectrode, and a Ag pseudoreference electrode.

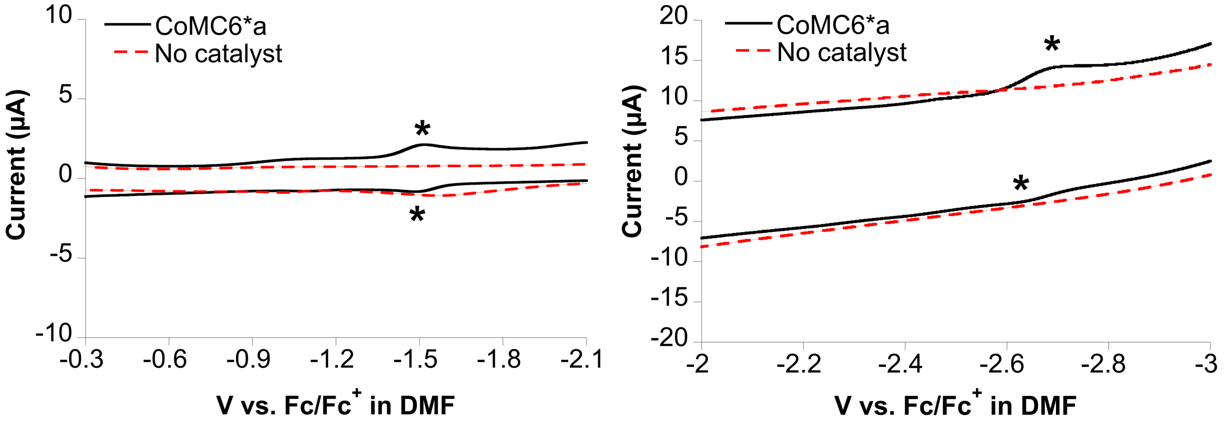

Fig. S17 The Co(III/II) and Co(II/II) couples recorded of 100 μM CoMC6*a observed by CV under air in DMF with 100 mM NBu_4PF_6 electrolyte. **Left panel:** The Co(III/II) (~–1.49 V vs. Fc/Fc^+) redox transition resolved at 50 mV/s scan rate. **Right panel:** The Co(II/I) (~–2.66 V vs. Fc/Fc^+) redox transition resolved at 500 mV/s scan rate. Peaks are marked with an asterisk (*). These experiments used a glassy carbon working electrode, a Pt counterelectrode, and a Ag pseudoreference electrode.

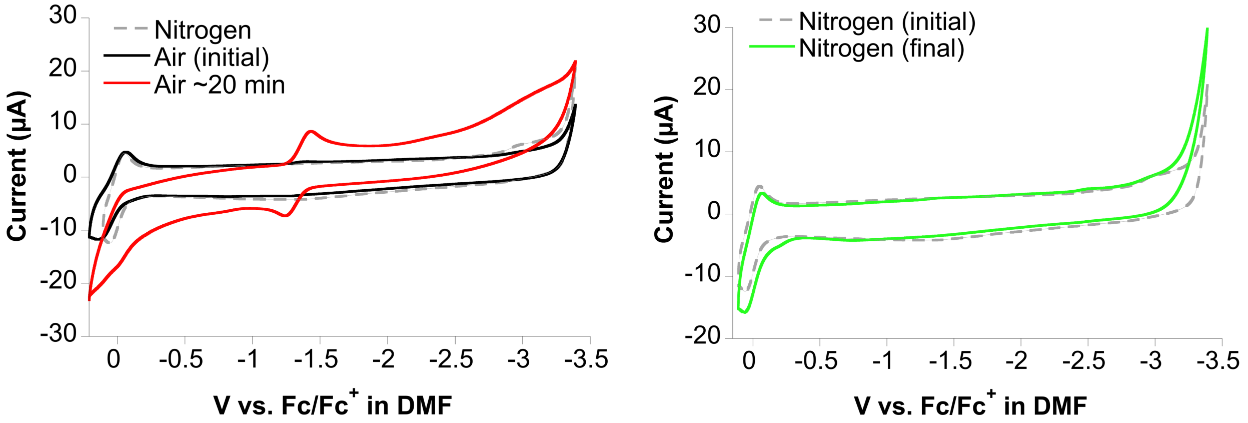

Fig. S18 Left panel: Cyclic voltammograms of 200 μM ferrocene in DMF with 100 mM NBu_4PF_6 electrolyte at a glassy carbon electrode taken inside an N_2-filled glovebox (dashed gray line). After exposing the sample to air for ~1-2 min (solid black line), the CV remains similar to the CV taken initially in the glovebox. After ~20 min, a reversible peak appears with a midpoint potential of −1.35 V vs. Fc/Fc^+. **Right panel**: After returning the 100 mM NBu_4PF_6 and 200 μM ferrocene in DMF to an N_2-filled glovebox, the reversible feature at −1.35 V vs. Fc/Fc^+ disappears, and the CV (solid green line) is nearly identical to the initial CV taken inside the glovebox before exposure to air (dashed gray line). The feature observed in air is attributed to the reversible reduction of O_2 to superoxide, which has been previously reported for glassy carbon electrodes.[6] These experiments used a Pt counterelectrode, a Ag pseudoreference electrode, and a scan rate of 500 mV/s.

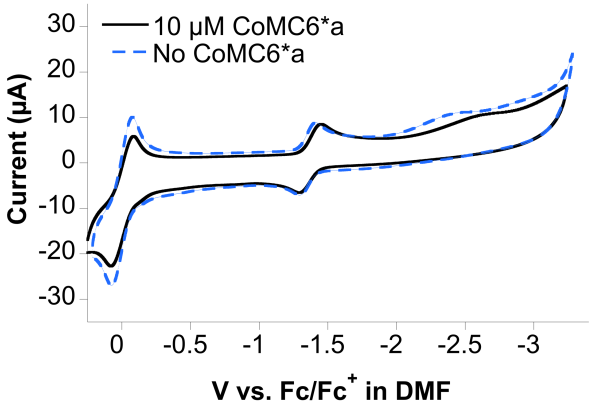

Fig. S19 Cyclic voltammogram of DMF and 100 mM NBu_4PF_6 electrolyte with (solid black line) and without (dashed blue line) 10 μM CoMC6*a in the presence of air. These experiments used a glassy carbon working electrode, a Pt counterelectrode, a Ag pseudoreference electrode, and a scan rate of 500 mV/s.

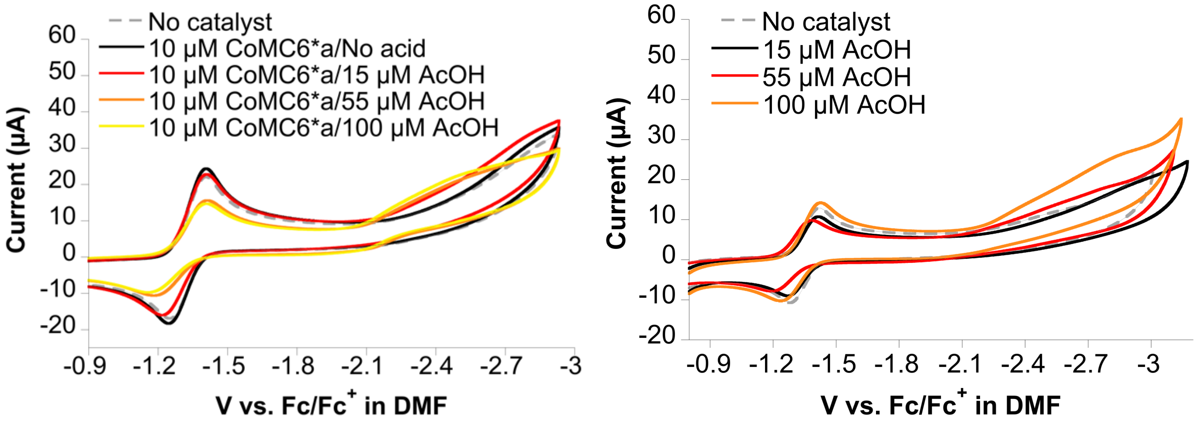

Fig. S20 Left panel: Cyclic voltammograms under air of DMF with 100 mM NBu_4PF_6 (dashed gray line), of 10 μM CoMC6*a in DMF with 100 mM NBu_4PF_6 (solid black line), and of 10 μM CoMC6*a and acetic acid (AcOH) at final AcOH concentrations of 15-100 μM. (red, orange yellow lines). **Right panel**: CVs under air of AcOH at final concentrations of 15, 55, and 100 μM (black, red, orange lines; no CoMC6*a added). These experiments used a glassy carbon working electrode, a Pt counterelectrode, a Ag pseudoreference electrode, and a scan rate of 500 mV/s.

References

1. R. M. Kellett and T. G. Spiro, *Inorg Chem*, 1985, **24**, 2378-2382.
2. J. G. Kleingardner, B. Kandemir and K. L. Bren, *J Am Chem Soc*, 2014, **136**, 4-7.
3. B. Kandemir, L. Kubie, Y. X. Guo, B. Sheldon and K. L. Bren, *Inorg Chem*, 2016, **55**, 1355-1357.
4. F. Lakadamyali, M. Kato, N. M. Muresan and E. Reisner, *Angew Chem Int Edit*, 2012, **51**, 9381-9384.
5. B. Mondal, K. Sengupta, A. Rana, A. Mahammed, M. Botoshansky, S. G. Dey, Z. Gross and A. Dey, *Inorg Chem*, 2013, **52**, 3381-3387.
6. P. S. Jain and S. Lal, *Electrochim Acta*, 1982, **27**, 759-763.
